# Plaque Rupture in Coronary Atherosclerosis Is Associated With Increased Plaque Structural Stress

**DOI:** 10.1016/j.jcmg.2017.04.017

**Published:** 2017-12

**Authors:** Charis Costopoulos, Yuan Huang, Adam J. Brown, Patrick A. Calvert, Stephen P. Hoole, Nick E.J. West, Jonathan H. Gillard, Zhongzhao Teng, Martin R. Bennett

**Affiliations:** a Division of Cardiovascular Medicine, University of Cambridge, Cambridge, United Kingdom; b Department of Radiology, University of Cambridge, Cambridge, United Kingdom; c Department of Interventional Cardiology, Papworth Hospital NHS Trust, United Kingdom; d Department of Engineering, University of Cambridge, Cambridge, United Kingdom

**Keywords:** atherosclerosis, coronary disease, vulnerable plaque, ACS, acute coronary syndrome(s), FA, fibroatheroma, FEA, finite element analysis, GS, gray-scale, IVUS, intravascular ultrasound, LAD, left anterior descending, MACE, major adverse cardiovascular event(s), MI, myocardial infarction, MLA, minimal luminal area, OCT, optical coherence tomography, PB, plaque burden, PCI, percutaneous coronary intervention, PSS, plaque structural stress, RCA, right coronary artery, TCFA, thin-cap fibroatheroma, VH, virtual histology

## Abstract

**Objectives:**

The aim of this study was to identify the determinants of plaque structural stress (PSS) and the relationship between PSS and plaques with rupture.

**Background:**

Plaque rupture is the most common cause of myocardial infarction, occurring particularly in higher risk lesions such as fibroatheromas. However, prospective intravascular ultrasound–virtual histology studies indicate that <10% higher risk plaques cause clinical events over 3 years, indicating that other factors also determine plaque rupture. Plaque rupture occurs when PSS exceeds its mechanical strength; however, the determinants of PSS and its association with plaques with proven rupture are not known.

**Methods:**

We analyzed plaque structure and composition in 4,053 virtual histology intravascular ultrasound frames from 32 fibroatheromas with rupture from the intravascular ultrasound–virtual histology in Vulnerable Atherosclerosis study and 32 fibroatheromas without rupture on optical coherence tomography from a stable angina cohort. Mechanical loading in the periluminal region was estimated by calculating maximum principal PSS by finite element analysis.

**Results:**

PSS increased with increasing lumen area (r = 0.46; p = 0.001), lumen eccentricity (r = 0.32; p = 0.001), and necrotic core ≥10% (r = 0.12; p = 0.001), but reduced when dense calcium was ≥10% (r = −0.12; p = 0.001). Ruptured fibroatheromas showed higher PSS (133 kPa [quartiles 1 to 3: 90 to 191 kPa] vs. 104 kPa [quartiles 1 to 3: 75 to 142 kPa]; p = 0.002) and variation in PSS (55 kPa [quartiles 1 to 3: 37 to 75 kPa] vs. 43 kPa [quartiles 1 to 3: 34 to 59 kPa]; p = 0.002) than nonruptured fibroatheromas, with rupture primarily occurring either proximal or immediately adjacent to the minimal luminal area (87.5% vs. 12.5%; p = 0.001). PSS was higher in segments proximal to the rupture site (143 kPa [quartiles 1 to 3: 101 to 200 kPa] vs. 120 kPa [quartiles 1 to 3: 78 to 180 kPa]; p = 0.001) versus distal segments, associated with increased necrotic core (19.1% [quartiles 1 to 3: 11% to 29%] vs. 14.3% [quartiles 1 to 3: 8% to 23%]; p = 0.001) but reduced fibrous/fibrofatty tissue (63.6% [quartiles 1 to 3: 46% to 78%] vs. 72.7% [quartiles 1 to 3: 54% to 86%]; p = 0.001). PSS >135 kPa was a good predictor of rupture in higher risk regions.

**Conclusions:**

PSS is determined by plaque composition, plaque architecture, and lumen geometry. PSS and PSS variability are increased in plaques with rupture, particularly at proximal segments. Incorporating PSS into plaque assessment may improve identification of rupture-prone plaques.

## Graphical abstract

**Figure:**
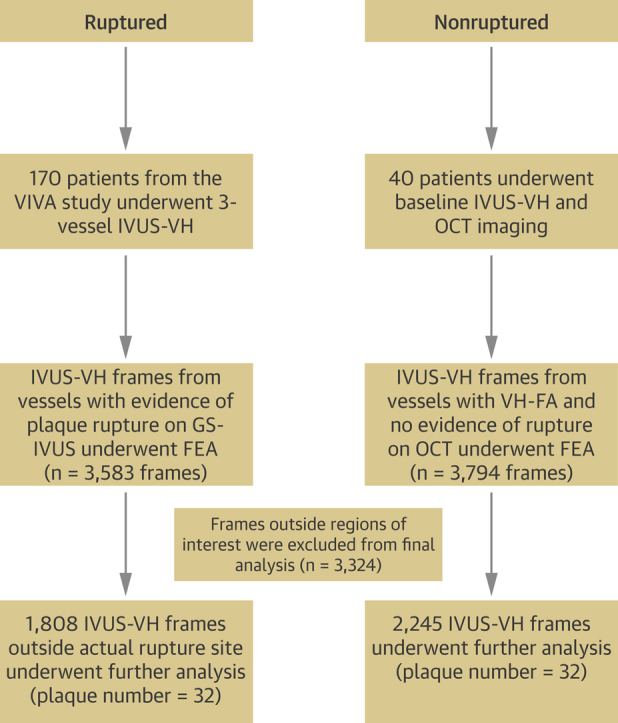

Rupture of a coronary plaque is the precipitating event in the majority of myocardial infarctions (MI) (1). Postmortem (2) and in vivo intravascular studies 3, 4 identify fibroatheromas (FAs), and in particular thin-cap fibroatheromas (TCFAs), as the most common predisposing lesion. TCFAs are widespread in human coronary artery disease, including asymptomatic individuals and those with stable and unstable syndromes (3). However, the incidence of major adverse cardiovascular events (MACEs) associated with TCFAs identified by intravascular ultrasound–virtual histology (IVUS-VH) is <10% over ∼3 years of follow-up 3, 4, suggesting that factors other than plaque and lumen size or plaque phenotype are important in determining plaque rupture.

Plaque rupture occurs when intraplaque stress exceeds the material strength of the overlying fibrous cap; increased plaque structural stress (PSS) is therefore a potential mechanism that determines rupture of a higher risk lesion. PSS can be calculated through an engineering technique known as finite element analysis (FEA), which approximates a solution to the equations of mechanical equilibrium by considering tissue material properties, plaque geometry, and local hemodynamic forces. Histological and IVUS-VH studies have identified necrotic core size, fibrous cap thickness, and the presence of microcalcification as important determinants of PSS 5, 6, 7. PSS has also been shown to be increased in patients presenting with acute coronary syndromes (ACS) versus stable symptoms (8). However, the determinants of PSS and its relationship to plaques that demonstrate rupture in vivo in human coronary arteries are not known. We sought to identify the parameters that determine PSS and variations in PSS across ruptured and nonruptured FAs identified using IVUS-VH to determine whether plaque stress is increased in plaques that have experienced rupture, and whether incorporating PSS into plaque assessment improves identification of rupture-prone plaques.

## Methods

### Study design

**Figure 1:**
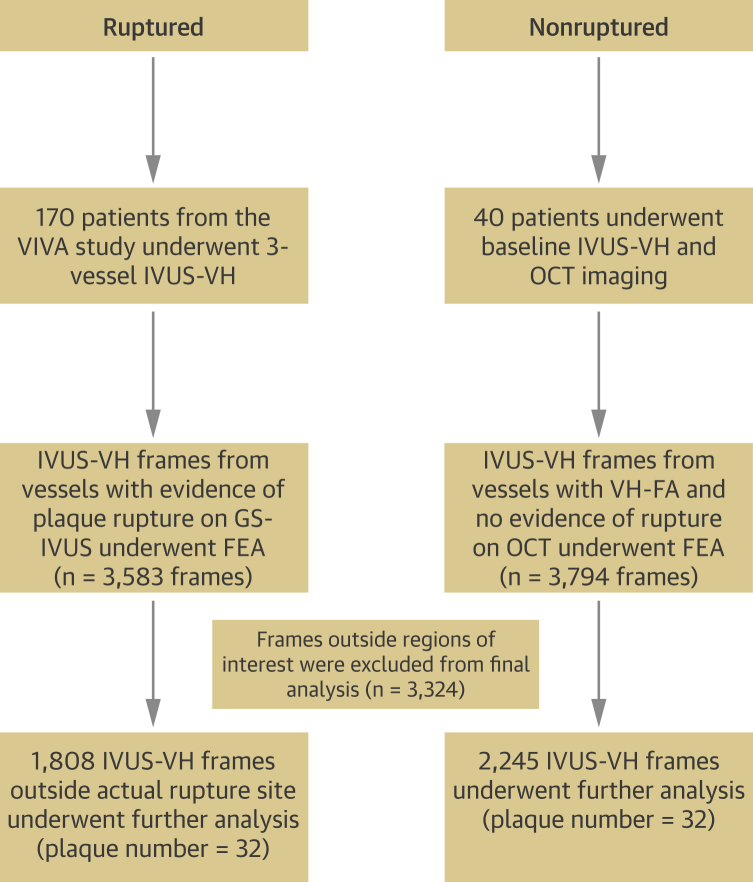
Schematic Representation of Patient and Plaque Populations Included in the Study FEA = finite element analysis; OCT = optical coherence tomography; VH-FA = virtual histology fibroatheroma; VIVA = VH-IVUS in Vulnerable Atherosclerosis study.

All plaques from the VIVA (VH-IVUS in Vulnerable Atherosclerosis) study with evidence of rupture (n = 32) on gray-scale (GS) IVUS were included in the study (3). The presence of rupture was verified by the Krakow Cardiovascular Research Institute core laboratory. To compare ruptured versus nonruptured plaques, we performed optical coherence tomography (OCT) before IVUS imaging on a separately recruited cohort of 40 patients with stable angina admitted for elective percutaneous coronary intervention (PCI) (Ethics Committee approval ref 11/EE/0277). OCT imaging ensured that only plaques with no evidence of rupture or erosion were included in the nonruptured control group. Because spontaneous rupture occurred only in FAs in VIVA, only IVUS-VH–identified FAs (VH-FAs) were included in the nonruptured group, yielding 32 plaques from 32 patients (Figure 1, Online Appendix).

### IVUS-VH and OCT analysis

**Figure 2:**
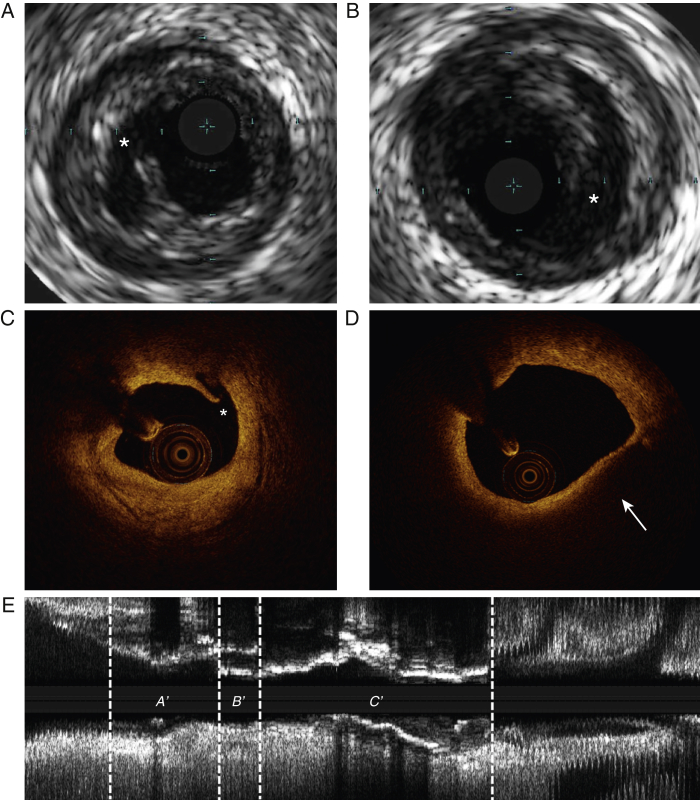
Identification of Plaque Rupture Using Gray-Scale Intravascular Ultrasound and Optical Coherence Tomography **(A and B)** Gray-scale IVUS images of spontaneous plaque rupture (*). **(C)** OCT image showing evidence of rupture (*).(D) Thin-cap fibroatheroma **(arrow)** as identified by OCT with no evidence of rupture or erosion. **(E)** Longitudinal IVUS reconstruction of a coronary artery with evidence of plaque rupture; proximal **(*A′*)**; rupture site **(*B′*)**; and distal **(*C′*)** segments. IVUS = intravascular ultrasound; other abbreviation as in Figure 1.

IVUS-VH data were acquired with 20-MHz Eagle-Eye catheters (Volcano Corporation, Rancho, Cordova) using motorized pullback at 0.5 mm/s. OCT data were acquired with Dragonfly C7 catheters (St. Jude Medical, St. Paul, Minnesota) using an automated pullback at 20 mm/s. Plaque classification, identification of rupture (Figure 2) and characterization of rupture location along the plaque length (Online Figure 1) were performed as previously described (Online Appendix). IVUS-VH plaque frames that demonstrated ruptures were not included in the final analysis for ruptured plaques, unless otherwise stated, because the extreme luminal eccentricity resulting from rupture at these frames would make PSS calculations unreliable.

### Biomechanical analysis

**Figure 3:**
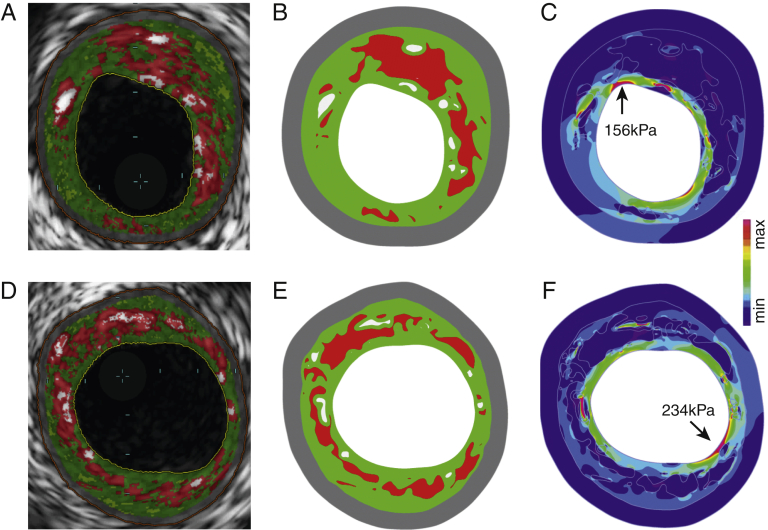
Illustrative Examples for Stepwise Calculation of Plaque Structural Stress From IVUS-VH Through Finite Element Analysis **(A and D)** IVUS-VH images showing necrotic core **(red)**, dense calcium **(white)**, fibrofatty tissue **(light green)**, and fibrous **(green)**. **(B and E)** Reconstructed plaque geometry and segmented plaque components used for finite element analysis. **(C and F)** Plaque structural stress band plots identifying regions with high stress concentration (**arrows** indicate areas of high stress at the plaque/lumen boundary). IVUS-VH = intravascular ultrasound-virtual histology.

Plaques underwent dynamic 2-dimensional FEA simulations as previously described (8) (Online Appendix). Maximum principal stress was used to indicate the critical mechanical conditions within the structure, referred as PSS. Variation in PSS refers to the difference in PSS between systole and diastole. A 65-μm layer of fibrous tissue was introduced during mesh generation to account for the limited axial resolution of IVUS-VH to detect a fibrous cap between lumen and necrotic core/dense calcium. Examples of PSS band plots with their corresponding IVUS-VH images are shown in Figure 3. Because PSS varies between frames, analysis was also performed after dividing plaques into 2-mm segments and averaging PSS across the IVUS-VH frames composing each segment. To estimate PSS at the exact site of rupture, the luminal boundary of frames demonstrating rupture was reconfigured with necrotic core present beneath this. PSS derived from these frames was compared with PSS from frames from the control cohort that demonstrated rupture after balloon inflation (Online Figure 2).

### Statistical analysis

Data were assessed for normality using the Shapiro-Wilk test. Normally distributed variables are presented as mean ± SD and compared using unpaired Student *t* test, and non-normal outcomes presented as median (quartiles 1 to 3) using Mann-Whitney *U* tests. Correlations between variables are expressed as Spearman correlation coefficients for non-normally distributed data. Generation of regression curves between PSS and plaque parameters was dependent on the value of the standard error of the estimate. Multivariable regression modeling was performed to determine the independent predictors of PSS with purposeful selection of covariates in the cohort of IVUS-VH frames of plaque burden (PB) ≥70% and necrotic core >10% because these have been linked with future events 3, 4, 9. Variables associated on univariate analysis (p < 0.10) were included in the multivariable model-building process. Because each plaque had multiple IVUS-VH slices, a linear mixed-effects model was used to compare groups using a random effect for plaque and fixed effects for group to account for clustering. All calculations were 2-tailed with p < 0.05 considered statistically significant. Statistical analyses were performed both in SPSS 19.0.0 (SPSS Inc., IBM Computing, Armonk, New York) and R 2.10.1 (The R Foundation for Statistical Computing, Vienna, Austria).

## Results

### Baseline patient and IVUS-VH demographics

We analyzed PSS in patients who had spontaneous plaque rupture on GS-IVUS from the VIVA study (n = 25 patients, n = 32 plaques) compared with patients undergoing elective PCI in which plaque rupture was excluded by both GS-IVUS and OCT (n = 32 patients, n = 32 plaques) (Figures 1 and 2). Patient demographics were similar between the ruptured and nonruptured groups (Table 1), with no differences in procedural systolic (129.9 ± 25.1 vs. 123.8 ± 18.9 mm Hg; p = 0.31) or diastolic blood pressure (66.2 ± 8.5 vs. 65.6 ± 9.3 mm Hg; p = 0.85).

**Table 1:** Demographics of Patients With Ruptured and Nonruptured Plaques

	Ruptured (n = 25)	Nonruptured (n = 32)	p Value
Age, yrs	61.4 ± 10.4	64.0 ± 10.1	0.46
Male	20.0 (80.0)	29.0 (90.6)	0.25
Diabetes mellitus	2.0 (8.0)	6.0 (18.8)	0.25
Hypertension	9.0 (36.0)	10.0 (31.3)	0.71
Hypercholesterolemia	8.0 (32.0)	6.0 (18.8)	0.25
Smoker	8.0 (32.0)	6.0 (18.8)	0.25
Family history of CAD	14.0 (56.0)	12.0 (37.5)	0.43
Procedural blood pressure			
Systolic BP, mm Hg	129.9 ± 25.1	123.8 ± 18.9	0.31
Diastolic BP, mm Hg	66.2 ± 8.5	65.6 ± 9.3	0.85
Total cholesterol, mmol/l	3.57 ± 0.88	3.83 ± 0.97	0.35
LDL cholesterol, mmol/l	2.37 ± 0.84	2.68 ± 0.98	0.25
HDL cholesterol, mmol/l	1.21 ± 0.47	1.15 ± 0.26	0.60
Total cholesterol:HDL ratio	3.34 ± 1.62	3.51 ± 1.47	0.72
Creatinine, mg/dl	85.6 ± 17.5	94.9 ± 22.5	0.11
hsCRP, mg/l	11.6 ± 28.9	5.36 ± 8.8	0.26

Values are mean ± SD or n (%).

BP = blood pressure; CAD = coronary artery disease; hsCRP = high-sensitivity C-reactive protein; HDL = high-density lipoprotein; LDL = low-density lipoprotein; MI = myocardial infarction.

There were no significant differences in plaque classification between the 2 groups, with VH-TCFA being the predominant plaque type accounting for rupture or undergoing PCI for stable angina (91 vs. 75%; p = 0.14) (Table 2). PB was lower in ruptured plaques (59.5% vs. 63.5%; p = 0.01), but minimal luminal area (MLA) and multiple components of plaque composition (% fibrous/fibrofatty tissue, necrotic core, dense calcium) were similar. Patient demographics and plaque composition for the cohort of patients with only VH-TCFAs also did not differ significantly between the groups (Online Tables 1 and 2).

**Table 2:** IVUS-VH Parameters of Ruptured and Nonruptured Plaques

	Ruptured (n = 32)	Nonruptured (n = 32)	p Value
VH-TCFA	29.0 (90.6)	24.0 (75.0)	0.14
Plaque burden	58.2 (6.8)	63.5 (4.8)	0.01
Plaque length, mm	22.4 ± 13.3	26.9 ± 16.5	0.17
Minimal luminal area ≤4 mm^2^	30.0 (93.8)	32.0 (100.0)	0.14
Fibrous/fibrofatty	60.8 (54.0–70.0)	67.5 (60.0–75.0)	0.10
Necrotic core, %	18.9 (15.0–27.0)	18.3 (14.0–23.0)	0.46
Dense calcium, %	15.8 (11.0–24.0)	14.1 (7.0–22.0)	0.33
Plaque characteristics at frame level			
Fibrous/fibrofatty, %	67.5 (50.0–82.0)	69.1 (50.0–84.0)	0.44
Necrotic core, %	16.5 (9.0–26.0)	15.6 (9.0–26.0)	0.18
Dense calcium, %	11.8 (4.0–24.0)	11.2 (4.0–25.0)	0.58
Maximum arc of fibrous/fibrofatty tissue, °	137.3 (93.0–205.0)	140.3 (94.0–205.0)	0.24
Maximum arc of necrotic core, °	38.5 (25.0–60.0)	37.1 (22.0–57.0)	0.18
Maximum arc of dense calcium, °	40.5 (18.0–76.0)	37.1 (18.0–64.0)	0.12
Lumen eccentricity	0.51 (0.4–0.6)	0.5 (0.4–0.6)	0.38

Values are n (%), mean ± SD, or median (quartiles 1 to 3).

IVUS-VH = intravascular ultrasound-virtual histology; VH-TCFA = virtual histology thin-cap fibroatheroma.

### Determinants of PSS in VH-defined fibroatheromas

We used FEA to estimate both the level and location of PSS in both ruptured and nonruptured plaques and the plaque and vessel determinants of PSS. Both luminal area and luminal eccentricity correlated positively with PSS (r = 0.46, p = 0.001; r = 0.32, p = 0.001, respectively), whereas a negative correlation was observed with PB (r = −0.23; p = 0.001) (Table 3). A positive, albeit weaker, correlation with PSS was also observed when frames with necrotic core ≥10% were considered (r = 0.12; p = 0.001) (Table 3), indicating that necrotic core affects PSS only when its contribution exceeds 10%. In contrast, dense calcium ≥10% and maximum arc of dense calcium demonstrated weak negative correlations with PSS (r = −0.12 and −0.13, respectively; both p = 0.001) (Table 3), suggesting that calcification may offer a plaque “shielding” effect when present in significant amounts. Fibrous/fibrofatty tissue was associated with PSS only when increasingly confluent, with no correlation seen with % tissue (r = 0.02; p = 0.13) (Table 3), but a weak negative correlation observed with maximum arc (r = −0.19; p = 0.001). PSS variability during the cardiac cycle was correlated with broadly similar parameters to peak PSS (Online Table 3), although fibrous/fibrofatty tissue (%) was negatively correlated and maximum arc of dense calcium was not correlated.

**Table 3:** Correlation Coefficients Between PSS and IVUS-VH Parameters

	Correlation Coefficient (r)	p Value
Luminal area, mm^2^	0.46	0.001
Plaque burden, %	−0.23	0.001
Lumen eccentricity	0.32	0.001
Necrotic core ≥10, %	0.12	0.001
Maximum arc of necrotic core, °	0.01	0.70
Dense calcium ≥10, %	−0.12	0.001
Maximum arc of dense calcium, °	−0.13	0.001
Fibrous/fibrofatty tissue, %	0.02	0.13
Maximum arc of fibrous/fibrofatty tissue, °	−0.19	0.001

PSS = plaque structural stress; other abbreviation as in Table 2.

**Figure 4:**
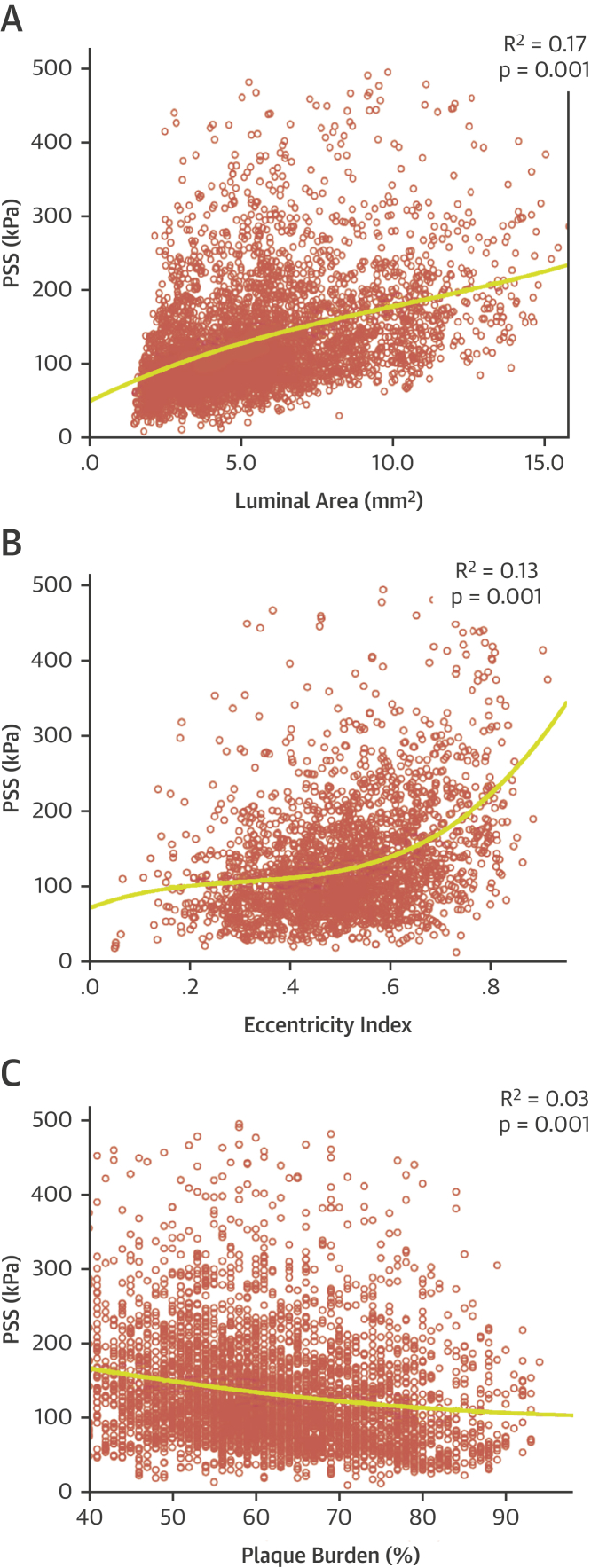
Indices of Vessel Geometry and Plaque Structural Stress **(A to C)** Regression curves illustrating best-fit relationships between PSS and **(A)** luminal area, **(B)** luminal eccentricity or **(C)** plaque burden. PSS = plaque structural stress.

**Figure 5:**
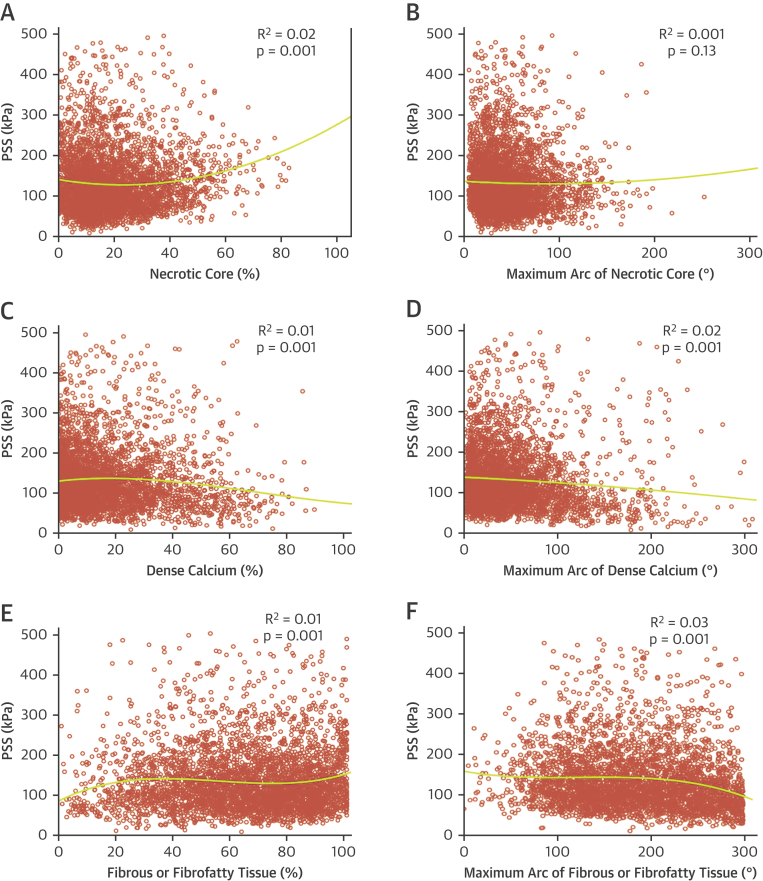
Effect of Plaque Components on PSS Regression curves illustrating best-fit relationships between PSS and **(A)** necrotic core (%), **(B)** maximal arc of necrotic core (°), **(C)** dense calcium (%), **(D)** maximal arc of dense calcium (°), **(E)** fibrous/fibrofatty tissue (%), and **(F)** maximal arc of fibrous/fibrofatty tissue (°). Abbreviation as in Figure 4.

Our data show that PSS is determined by a number of factors, which differ depending on plaque type and extent of disease. We therefore undertook regression analysis to assess whether individual plaque parameters could predict PSS in FAs. This demonstrated that neither luminal area, plaque eccentricity, PB (Figures 4A to 4C), nor plaque composition (Figures 5A to 5F) could individually determine PSS.

PSS is likely to have the greatest clinical impact when high stress occurs in a higher risk region. We therefore used multivariable regression modeling to identify independent determinants of PSS in higher risk segments. Luminal area, PB, % necrotic core, % dense calcium, fibrous/fibrofatty tissue arc, necrotic core arc, calcium arc, and lumen eccentricity were all included in the final model. A significant regression equation was observed (F [8,677] = 82.1; p = 0.001), with an R^2^ of 0.49 (R^2^_adjusted_ = 0.49). Luminal area (β = 0.53; p = 0.001), necrotic core arc (β = 0.10; p = 0.04), and lumen eccentricity (β = 0.32; p = 0.001) were independent positive predictors of PSS, and calcium arc (β = −0.12; p = 0.003) and fibrous/fibrofatty tissue arc (β = −0.25; p = 0.001) were independent negative predictors of PSS.

### PSS and plaque rupture

**Figure 6:**
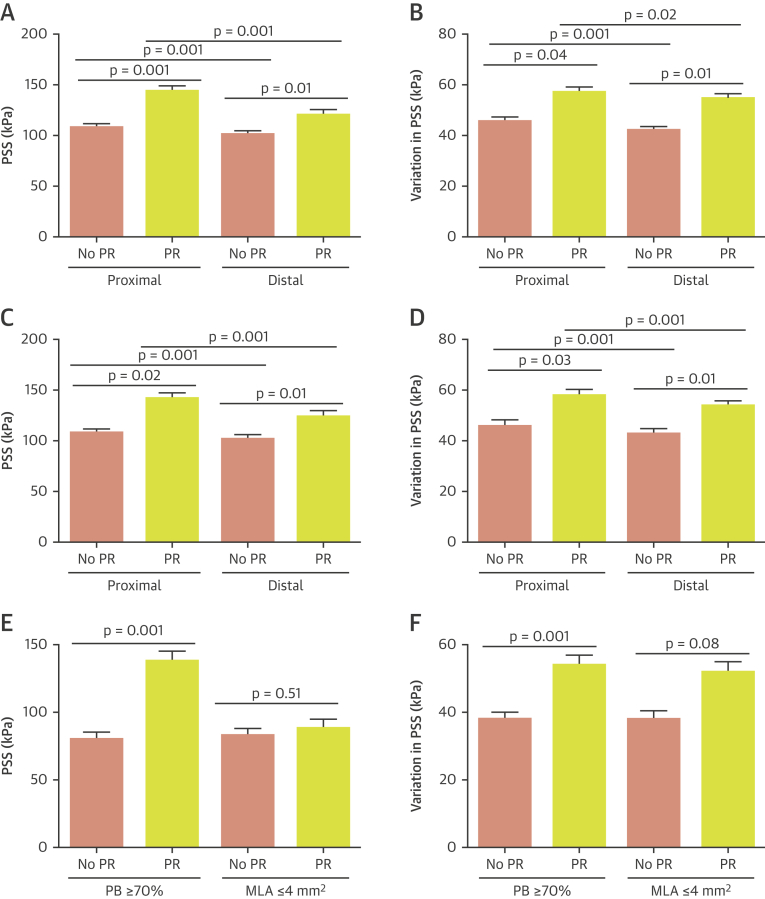
PSS and Variation in PSS in VH-FAs With and Without Rupture **(A and B)** PSS and variation in PSS in the proximal and distal segments of VH-FAs with and without plaque rupture. **(C and D)** PSS and variation in PSS in the proximal and distal segments of VH-TCFAs with and without PR. **(E and F)** PSS and variation in PSS in VH-TCFAs with PB ≥70% or MLA ≤4 mm^2^ with and without PR. MLA = minimal luminal area; PR = plaque rupture; VH-TCFA = virtual histology thin-cap fibroatheroma; other abbreviations as in Figures 1 and 4.

Although our data indicate that PSS is associated with plaque features considered to promote rupture, the relationship between PSS and plaques that demonstrate actual rupture is unclear. We therefore examined PSS in plaques with GS-IVUS evidence of rupture compared with plaques where rupture was excluded on OCT (Figure 2). Compared with nonruptured FAs, ruptured FAs showed higher PSS (133 kPa [quartiles 1 to 3: 90 to 191 kPa] vs. 104 kPa [quartiles 1 to 3: 75 to 142 kPa]; p = 0.002) (Online Figure 3A); this was also seen when either proximal (143 kPa [quartiles 1 to 3: 101 to 200 kPa] vs. 108 kPa [quartiles 1 to 3: 78 to 150 kPa]; p = 0.001) or distal segments (120 kPa [quartiles 1 to 3: 78 to 180 kPa] vs. 101 kPa [quartiles 1 to 3: 71 to 138 kPa]; p = 0.01) were specifically examined (Figure 6A). Similarly, compared with nonruptured FAs, ruptured FAs showed greater variation in PSS between systole and diastole (55 kPa [quartiles 1 to 3: 37 to 75 kPa] vs. 43kPa [quartiles 1 to 3: 34 to 59 kPa]; p = 0.02) (Online Figure 3B); this was also seen when proximal (57 kPa [quartiles 1 to 3: 38 to 74 kPa] vs. 45 kPa [quartiles 1 to 3: 35 to 60 kPa]; p = 0.04) or distal (54 kPa [quartiles 1 to 3: 34 to 75 kPa] vs. 42 kPa [quartiles 1 to 3: 32 to 57 kPa]; p = 0.01) segments were examined (Figure 6B). Similar results were obtained when PSS was averaged across 2-mm segments (Online Figures 4A to 4C). An analysis focusing only on the 4-mm segments proximal and distal to the rupture site (ruptured group) and MLA (nonruptured group) also found PSS to be higher in plaques that experienced rupture (127 kPa [quartiles 1 to 3: 84 to 188 kPa] vs. 112 kPa [quartiles 1 to 3: 79 to 152 kPa]; p = 0.03) (Online Figures 2 and 4D to 4E).

**Figure 7:**
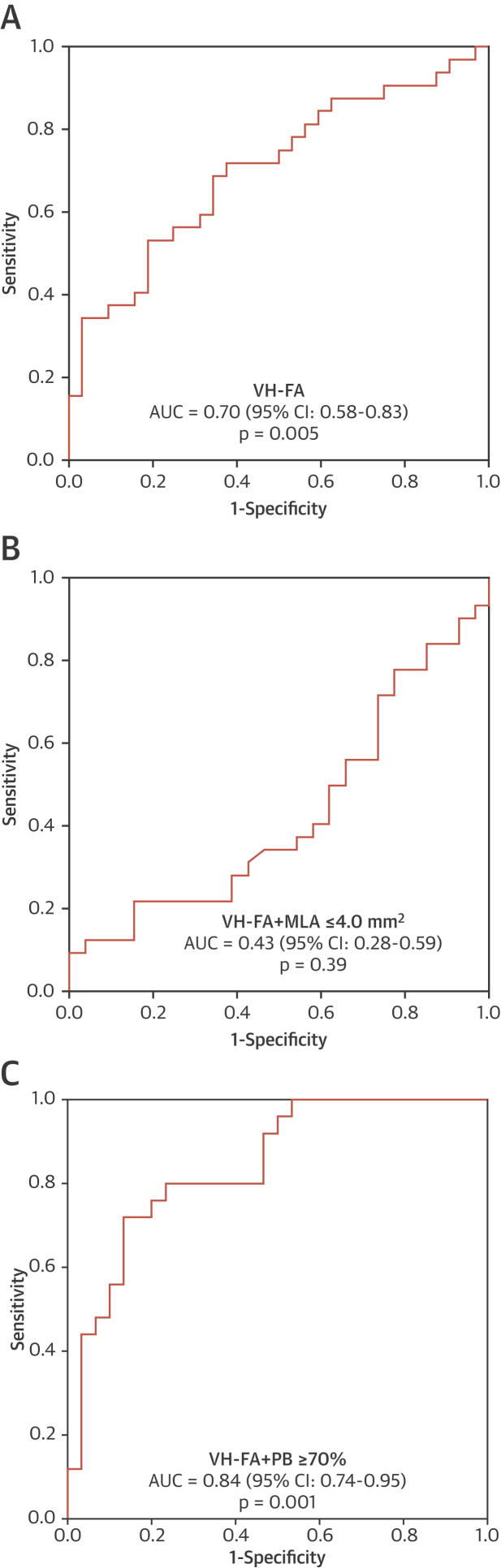
ROC Curves Illustrating the Discriminatory Power of PSS to Identify Plaque Rupture **(A)** ROC curve for PSS in VH-FA. **(B)** ROC curve for PSS in VH-FA + MLA ≤4 mm^2^. **(C)** ROC curve for PSS in VH-FA+PB ≥70%. AUC = area under the curve; CI = confidence interval; MLA = minimal lumen area; PB = plaque burden; ROC = receiver-operating characteristic; other abbreviation as in Figure 1.

Certain IVUS-VH features have been associated with a higher MACE risk, notably VH-TCFA, PB ≥70%, and MLA ≤4 mm^2^, and further increased by combinations of these features 3, 4, 9. PSS and variation in PSS were significantly higher in ruptured versus nonruptured VH-TCFAs (Figures 6C and 6D), and again this was seen in both proximal and distal segments. Similarly, both PSS and variation in PSS were higher in plaques that were VH-TCFAs with PB ≥70% (PSS: 137 kPa [quartiles 1 to 3: 95 to 190 kPa] vs. 80 kPa [quartiles 1 to 3: 57 to 113 kPa]; p = 0.001; variation in PSS: 54 kPa [quartiles 1 to 3: 38 to 74 kPa] vs. 37 kPa [quartiles 1 to 3: 28 to 49 kPa]; p = 0.001) (Figures 6E and 6F), but similar between ruptured and nonruptured VH-TCFAs when MLA ≤4 mm^2^ (PSS: 88 kPa [quartiles 1 to 3: 62 to 134 kPa] vs. 83 kPa [quartiles 1 to 3: 59 to 122 kPa]; p = 0.51; variation in PSS: 52 kPa [quartiles 1 to 3: 33 to 72 kPa] vs. 38 kPa [quartiles 1 to 3: 30 to 52 kPa]; p = 0.08) (Figures 6E and 6F). We also performed receiver operating characteristic curve analysis to assess the ability of PSS to predict plaque rupture (Figures 7A to 7C). Inclusion of PSS significantly improved the ability of the combination of VH-FA + PB ≥70% to identify plaque rupture (Figure 7C). An optimal PSS cutoff of 135 kPa was identified for this cohort, associated with good sensitivity (72.1%), specificity (83.3%), positive predictive (78.3%), and negative predictive (78.1%) values. Because PSS from ruptured frames was excluded from these analyses for reasons previously mentioned, attempts were made to estimate PSS at the site of rupture. This necessitated reconfiguration of the luminal boundary with the rupture cavity now occupied by necrotic core, which itself separated from the lumen with a 65-μm layer of fibrous tissue. PSS (140 kPa [quartiles 1 to 3: 96 to 199 kPa] vs. 85 kPa [quartiles 1 to 3: 60 to 125 kPa]; p = 0.004) and variation in PSS (76 kPa [quartiles 1 to 3: 56 to 95 kPa] vs. 47 kPa [quartiles 1 to 3: 37 to 63 kPa]; p = 0.01) from these sites were higher compared with frames from the nonruptured cohort that demonstrated rupture only after balloon inflation (Online Figure 4F).

### Location of plaque rupture and distribution of PSS in ruptured plaques

We found that ruptures were more common in the right coronary artery (RCA) (59.3%), followed by left anterior descending (LAD) (40.1%) and left circumflex (15.6%) arteries, with 80% of ruptures occurring in the first 33 mm of the artery. Rupture occurred primarily either in the proximal or middle (MLA and peri-MLA) plaque segments (87.5% vs. 12.5%; p = 0.001), in agreement with previously published data (10).

PSS and variations in PSS differed markedly over short distances both within the same plaque and between plaques, irrespective of whether these ruptured (Online Figure 5). We therefore examined whether differences in PSS could explain the location of rupture and whether this reflected differences in plaque composition. Both PSS (143 kPa [quartiles 1 to 3: 101 to 200 kPa] vs. 120k Pa [quartiles 1 to 3: 78 to 180 kPa]; p = 0.001) and variation in PSS (57 kPa [quartiles 1 to 3: 38 to 74 kPa] vs. 54 kPa [quartiles 1 to 3: 34 to 75 kPa]; p = 0.02) were higher in the segments proximal to rupture sites (Figures 6A and 6B, Online Figure 6) versus distal sites. Proximal plaque segments were composed of less fibrous/fibrofatty tissue (63.6% [quartiles 1 to 3: 46% to 78%] vs. 72.7% [quartiles 1 to 3: 54% to 86%]; p = 0.001) but greater necrotic core (19.1% [quartiles 1 to 3: 11% to 29%] vs. 14.3% [quartiles 1 to 3: 8% to 23%]; p = 0.001) and dense calcium (13.2% [quartiles 1–3: 6% to 25%] vs. 10.1% [quartiles 1 to 3: 3% to 23%]; p = 0.001) than distal sites. Necrotic core arc (43° [quartiles 1 to 3: 27 to 66°] vs. 34° [quartiles 1 to 3: 21 to 53°]; p = 0.001), calcium arc (47° [quartiles 1 to 3: 23 to 81°] vs. 32° [quartiles 1 to 3: 14 to 66°]; p = 0.001) and fibrous/fibrofatty arc (144° [quartiles 1 to 3: 96 to 216°] vs. 131° [quartiles 1 to 3: 89 to 191°]); p = 0.001) were all also increased in the proximal segments. PSS in the LAD/RCA arteries was higher compared with the left circumflex (136 kPa [quartiles 1 to 3: 93 to 195 kPa] vs. 90 kPa [quartiles 1 to 3: 66 to 130 kPa]; p = 0.01), with no differences noted in PSS variation between the 2 vessel groups (54 kPa [quartiles 1 to 3: 36 to 74 kPa] vs. 64 kPa [quartiles 1 to 3: 45 to 80 kPa]; p = 0.18).

## Discussion

Plaque rupture is the precipitating event in the majority of MIs, and is thought to occur at sites of plaque weakness (1). PSS is a potential trigger of rupture, with rupture occurring when PSS exceeds the mechanical strength of the overlying fibrous cap. Ex vivo carotid studies have shown that peak circumferential stress is highest at areas of rupture (5), which has been confirmed by subsequent in vivo magnetic resonance imaging studies, despite the limited capability of magnetic resonance imaging scans to accurately identify plaque components (11); however, estimation of PSS in human coronary plaques in vivo is more challenging. Recently, we demonstrated that PSS estimated from IVUS-VH is higher in culprit plaques of patients presenting with ACS than stable symptoms (8); however, patients with ACS have a variety of plaque substrates and circumstances that account for their presentation. The current study examined the relationship between IVUS-VH–derived PSS and proven plaque rupture compared with plaques of similar classification in which rupture was excluded by OCT.

### PSS and plaque architecture

We find that PSS in coronary FAs is determined by multiple parameters, including plaque size, composition, and luminal geometry. PSS is increased by luminal area and eccentricity, but reduced by PB. PSS appears to be increased when necrotic core is ≥10%, and reduced when dense calcium ≥10%. However, these relationships are complex, and none of the individual plaque parameters examined showed strong correlations or were able to independently determine PSS, even when considered only in higher risk regions. The proximal segment of plaques also showed differences in composition that increase PSS compared with distal segments, including increased % necrotic core and decreased % fibrous/fibrofatty tissue. The observation that luminal area correlates positively with PSS indicates that plaques causing mild or moderate stenosis may be under greater stress and therefore more prone to rupture compared with plaques that cause greater lumen stenosis. This is consistent with serial coronary angiography studies that demonstrate that vessel occlusion, largely resulting from plaque rupture, often occurs at sites with less severe stenosis 12, 13, 14. Collectively, these results suggest that PSS is influenced by factors other than overall plaque composition, such as the spatial relationship between adjacent components and the lumen, parameters that are ignored by current classification systems of plaque vulnerability.

### PSS and plaque rupture

The frequency of plaque rupture varies between the main coronary arteries, with LAD and RCA FAs being more prone to rupture than left circumflex FAs, a similar pattern to coronary artery occlusion leading to ST-segment elevation MI 15, 16. In addition, FAs in the proximal segments are particularly at risk 15, 17. We find a similar pattern with rupture clustering in the RCA and LAD, and close to the coronary ostia, especially in the LAD 15, 17, similar to postmortem studies (17). We also find that proximal or peri-MLA plaque segments are more prone to rupture (10), and are sites expected to have higher PSS because of their larger luminal area, in accordance with Laplace’s law (σ = Pr/h, where σ = circumferential stress, P = intra-arterial pressure, r = vessel radius, and h = vessel wall thickness). Plaque segments proximal to the rupture site also showed higher PSS, and this was associated with significant differences in plaque composition. More importantly, we demonstrate that both PSS and variation in PSS are higher in ruptured than nonruptured plaques**,** which was also seen when only VH-TCFAs or PB ≥70% were examined. This remained when PSS was averaged over 2-mm plaque segments and when only the areas adjacent to rupture were examined. PSS varies throughout the cardiac cycle, with highest and lowest values corresponding to systolic and diastolic pressures. This cyclical stretching and relaxation may be particularly important to induce plaque “fatigue.” In agreement with this, ruptured FAs demonstrate greater variation in PSS both across the entire plaque length and in regions close to the site of rupture. This suggests that rupture occurs in a subset of higher risk plaques already under high stress, when either a further increase in PSS occurs or large variations in PSS promote cap fatigue (18), or both. PSS in the vicinity of 135 kPa appears to be particularly important, a cutoff that is associated with good positive and negative predictive values in identifying plaques that demonstrate rupture. Interestingly, following lumen reconstruction of frames with rupture, overall PSS was found to be greater than this cutoff, which also exceeded that from nonruptured frames that experienced rupture following balloon inflation. The finding that PSS did not differ between ruptured versus nonruptured plaques at sites of MLA ≤4 mm^2^ is consistent with results from the ATHEROREMO-IVUS (European Collaborative Project on Inflammation and Vascular Wall Remodeling in Atherosclerosis – Intravascular Ultrasound) study, in which MLA ≤4 mm^2^ did not predict future MACE (9).

### Study limitations

First, although we demonstrated that ruptured plaques were associated with higher PSS, we cannot calculate exact PSS levels at the precise rupture site. Our simulations suggest, however, that PSS at the site of rupture is likely to exceed 135 kPa, which also appears to be higher compared with segments prone to balloon-induced rupture. Second, because PSS calculations are dependent on the resolution of IVUS-VH and its ability to accurately identify plaque components, PSS derived from IVUS-VH has the same limitations. Nonetheless, IVUS-VH remains the only intravascular imaging modality with: 1) prospective clinical data correlating MACE with particular plaque phenotypes; 2) automatic component identification and segmentation, thus eliminating an important source of human error; and 3) sufficient penetration to image the whole plaque, which is important for accurate PSS calculations. Third, our study consists of 2 cohorts that were obtained at different time points. However, OCT was not performed in the VIVA study, but was necessary in the unruptured patient population to exclude rupture, thereby ensuring that the nonruptured group truly consisted of intact plaques.

## Conclusions

We demonstrate that IVUS-VH–based PSS is dependent on components of plaque composition and plaque and lumen architecture that are also associated with plaque vulnerability and rupture, thereby indirectly linking PSS to plaque rupture. More importantly, PSS is higher in plaques with proven rupture, particularly in segments proximal to the rupture site, which have increased necrotic core. PSS >135 kPa appears to be a good predictor of rupture in higher risk regions. Our results suggest that incorporation of PSS calculations into coronary plaque assessment may improve our ability to identify those plaques that proceed to rupture and clinical events.

Perspectives **COMPETENCY IN MEDICAL KNOWLEDGE:** PSS is determined by plaque composition as well as plaque and lumen architecture. PSS appears to be important in determining rupture, as in plaques with similar classification and composition, PSS is higher in those with rupture. PSS >135 kPa accurately identifies rupture suggesting that FAs in vivo with levels beyond this are at particular risk of such events.

**TRANSLATIONAL OUTLOOK:** Plaque rupture is the precipitating event in the majority of MIs; however, in prospective IVUS-VH studies, <10% of high-risk plaques were responsible for cardiac events over 3 years, suggesting that other factors play a role in this process. PSS can be estimated from IVUS-VH images, and is proposed as a mechanism that determines rupture in high-risk regions. Incorporation of PSS into assessment of coronary atherosclerotic plaques may improve our ability to identify those that cause clinical events.
