# Supplementary material for: Plaque Rupture in Coronary Atherosclerosis Is Associated With Increased Plaque Structural Stress
Source: JACC Cardiovasc Imaging. 2017 Dec;10(12):1472–83. doi: 10.1016/j.jcmg.2017.04.017 (PMC5725311; doi:10.1016/j.jcmg.2017.04.017)
Supplement: Online Data [file mmc1.pdf]

Online Supplement

Plaque rupture in coronary atherosclerosis is associated with increased plaque structural stress

Charis Costopoulos MD[1], Yuan Huang PhD, MD[2], Adam J. Brown MD, PhD[1], Patrick A. Calvert MD, PhD[4], Stephen P. Hoole MD[4], Nick E. J. West MD[4], Jonathan H. Gillard MD[2], Zhongzhao Teng PhD[2, 3], Martin R. Bennett, MD, PhD[1]

Methods

Study design

We examined images from the VH-IVUS in Vulnerable Atherosclerosis (VIVA) study to identify spontaneous plaque rupture on grey-scale IVUS (GS-IVUS). The VIVA study protocols were approved by the Cambridge Research Ethics Committee and have been described previously[1] (ref 07/Q0106/47). Briefly, following informed consent, 170 patients undergoing percutaneous coronary intervention (PCI) for either stable angina pectoris or acute coronary syndrome underwent 3-vessel virtual-histology intravascular ultrasound (VH-IVUS). To compare ruptured vs. non-ruptured plaques, we performed OCT (optical coherence tomography) prior to VH-IVUS imaging on a separately recruited cohort of 40 patients admitted for elective percutaneous coronary intervention (ref 11/EE/0277). Patients with previous revascularization, active malignancy, chronic renal failure (eGFR <30ml/min), hemodynamic instability or coronary anatomy unsuitable for invasive imaging were excluded from both groups.

VH-IVUS and OCT analysis

A plaque was defined when plaque burden (PB) was ≥40% of vessel cross-sectional area for 3 consecutive IVUS frames. Virtual-histology fibroatheromas (VH-FAs) were defined as plaques with confluent necrotic core >10% in 3 consecutive frames, and virtual-histology

thin-cap fibroatheromas (VH-TCFAs) as VH-FAs with a confluent necrotic core in contact with lumen, also in 3 consecutive frames. Plaque rupture on GS-IVUS was defined as a ruptured capsule with an underlying cavity or plaque excavation by atheromatous extrusion with no visible capsule and by optical coherence tomography (OCT) as a visible tear in the fibrous cap[2-4]. Sites of spontaneous rupture were identified in-house by two independent operators and verified by the Krakow Cardiovascular Research Institute core laboratory. Plaques with evidence of rupture separated by at least 5mm length of rupture-free vessel were considered as separate plaques. To examine the location of rupture along the plaque, each plaque was divided into 3 longitudinal portions (proximal, middle, distal) as previously described **(Fig. 1 in Online Appendix)**[5].

Study Definitions

Luminal eccentricity (eccentricity index) was defined as the ratio of the distance between the foci of the ellipse and its major axis length (an ellipse whose eccentricity is 0 is a circle, while an ellipse whose eccentricity is 1 is a line segment). Comparisons of plaque structural stress (PSS) and variation in PSS were performed across the entire plaque and between proximal and distal segments. In the ruptured group, proximal and distal segments refer to plaque regions proximal or distal to the site of spontaneous rupture. Proximal and distal segments in the non-ruptured group refer to segments proximal or distal to the minimal lumen area (MLA)(**Figure 1 in Online Appendix**).

Biomechanical Analysis

Plaque geometry was constructed from VH-IVUS data using an in-house MATLAB code (D3Plaque, Cambridge, UK). Any frames where PB<40% were excluded from biomechanical analysis, as these were considered non-atherosclerotic regions. As *in vivo* data are recorded during diastole, circumferential shrinkage was applied to generate a zero-pressure condition

for computational simulation as previously described[6,7]. Plaque components were assumed to be incompressible, piecewise homogeneous, non-linear isotropic and hyper-elastic as described by the modified Mooney-Rivlin strain energy density function:

$$W = c_1(\bar{I}_1 - 3) + D_1\left[e^{D_2(\bar{I}_1 - 3)} - 1\right] + \kappa(J - 1)$$

where $\bar{I}_1 = J^{-2/3} I_1$ with I_1 being the first invariant of the unimodular component of the left Cauchy-Green deformation tensor. $J = det(\boldsymbol{F})$ and $\boldsymbol{F}$ is the deformation gradient. κ is the Lagrangian multiplier for the incompressibility. c_1, D_1 and D_2 are material parameters derived from previous experimental work[8] and include; arterial vessel wall, c_1=0.138 kPa, D_1=3.833 kPa, D_2=18.803; fibrous tissue, c_1=0.186 kPa, D_1=5.769 kPa, D_2=18.219 and necrotic core, c_1=0.046 kPa, D_1=4.885 kPa, D_2=5.426. The material properties of dense calcification were derived by fitting a Young's modulus of 184 MPa derived from experimental work[9]: c_1=1.147×10^5 kPa, D_1=7.673×10^4 kPa and D_2=2.838×10^{-8}. The motion of each atherosclerotic component is governed by kinetic equations as:

$$\rho v_{i,tt} = \sigma_{ij,j} \ (i, j = 1, 2)$$

where $[v_i]$ and $[\sigma_{ij}]$ are the displacement vector and stress tensor, respectively, ρ is the density of each component and t stands for time.

The entire plaque geometric model was meshed using 9-node quadrilaterals (generating approximately 10,000 elements and 40,000 nodes per model). Displacement and strain were assumed to be large. There was no relative movement at the interface of atherosclerotic components and the relative energy tolerance was set to be 0.005. Two adjacent points were fixed to prevent rigid body displacement. PSS was used to characterize the mechanical loading within the plaque structure in the peri-luminal region (0.2mm maximum depth from the luminal contour). The choice of 0.2mm was influenced by the axial resolution of IVUS, the fact that plaque rupture is thought to be the result of superficial plaque destabilization and

by previous unpublished work that demonstrated that superficial but not deep PSS is capable of predicting future adverse events. Stress variation during one cardiac cycle was defined as:

$$\text{Variation of PSS} = max(PSS_i^t) - min(PSS_i^t)$$

in which the subscript *i* stands for the ith integration node and the superscript *t* stands for time were computed. Dynamic loading conditions were generated from coronary pressure recordings taken at the time of the procedure. Pressure at the outer boundary was set to zero. We initially analyzed 7,377 VH-IVUS frames by finite element analysis (FEA) but excluded frames outside plaques of interest as well as frames at the actual rupture site, as the tissue that had comprised the rupture site could not be assessed. The final analysis therefore compared 1,808 frames from ruptured and 2,245 frames from non-ruptured plaques (n=32 plaques in each group).

Supplemental Figure Legends

Figure 1. Segmentation of plaques into proximal, middle and distal segments for analysis of rupture sites.

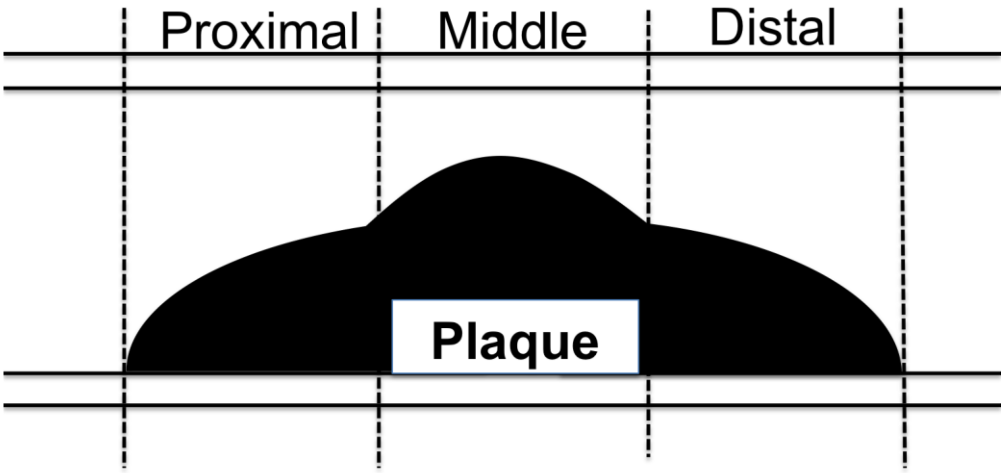

Figure 2. Segmentation of non-ruptured (A) and ruptured (B) plaques to 4mm segments proximal and distal to the rupture site/MLA. (*) indicates area bridged with necrotic core when estimating PSS at site of rupture.

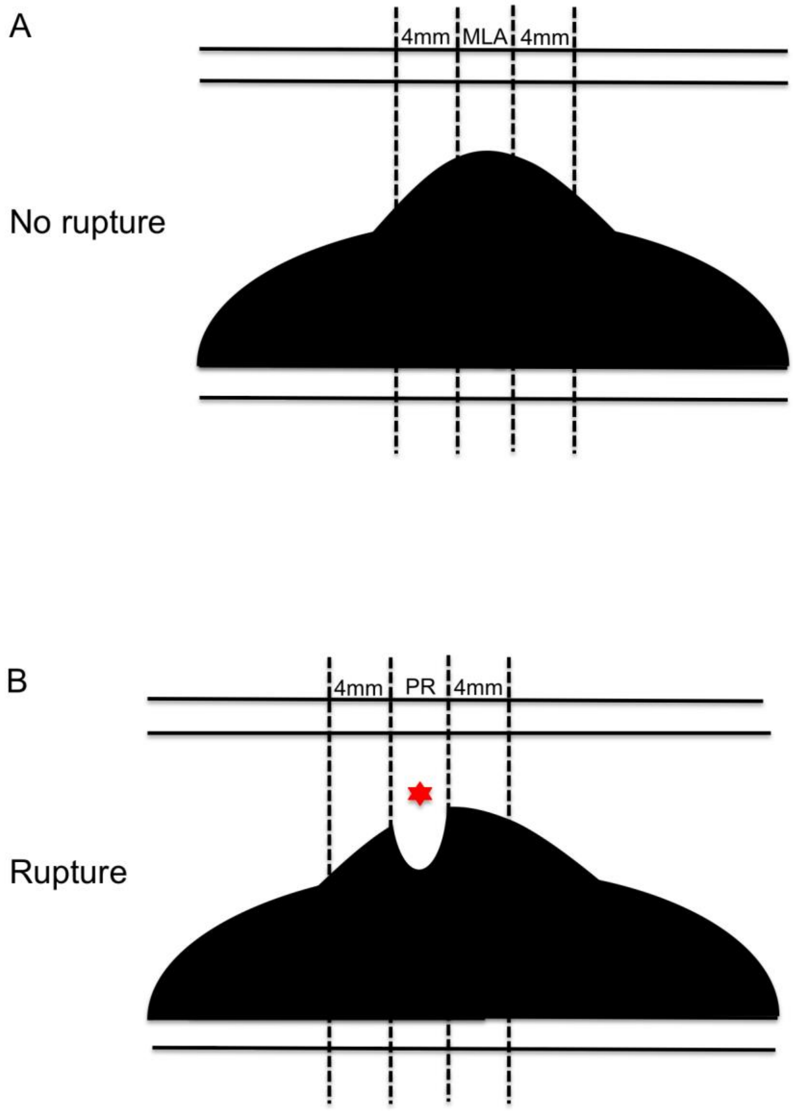

Figure 3. Distribution graphs for PSS (A) and variation in PSS (B) in ruptured and non-ruptured plaques.

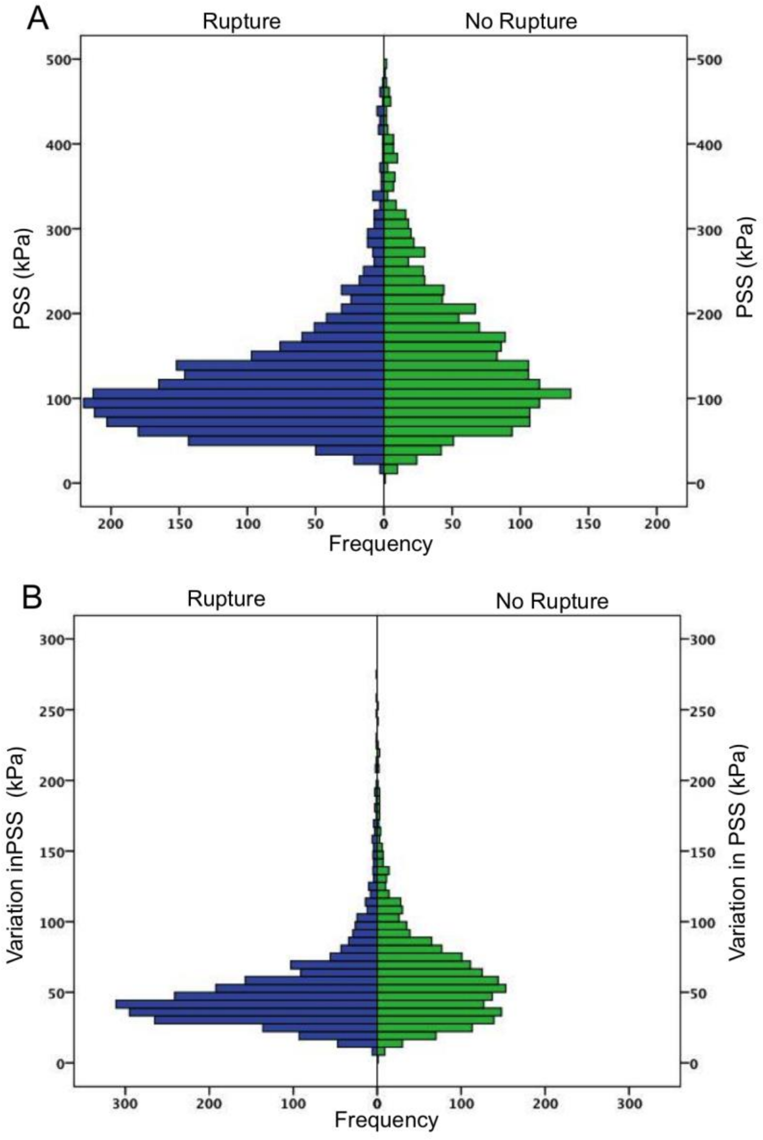

Figure 4. Plaque structural stress and variation in PSS in virtual-histology fibroatheromas with and without rupture (A-C) PSS and variation in PSS across VH-FA across the whole plaque (A), proximal (B) and distal (C) sites after dividing plaques in 2mm segments. (D and E) PSS and variation in PSS across either both proximal and distal 4mm segments (D) or only proximal 4mm segments (E) to the PR or MLA site (as in Online Fig. 2) (F) PSS and variation in PSS in frames with rupture (following lumen re-configuration) and frames from the non-ruptured cohort that ruptured after balloon inflation MLA=minimal luminal area; PR=plaque rupture; PSS=plaque structural stress; VH-FA=virtual-histology fibroatheroma.

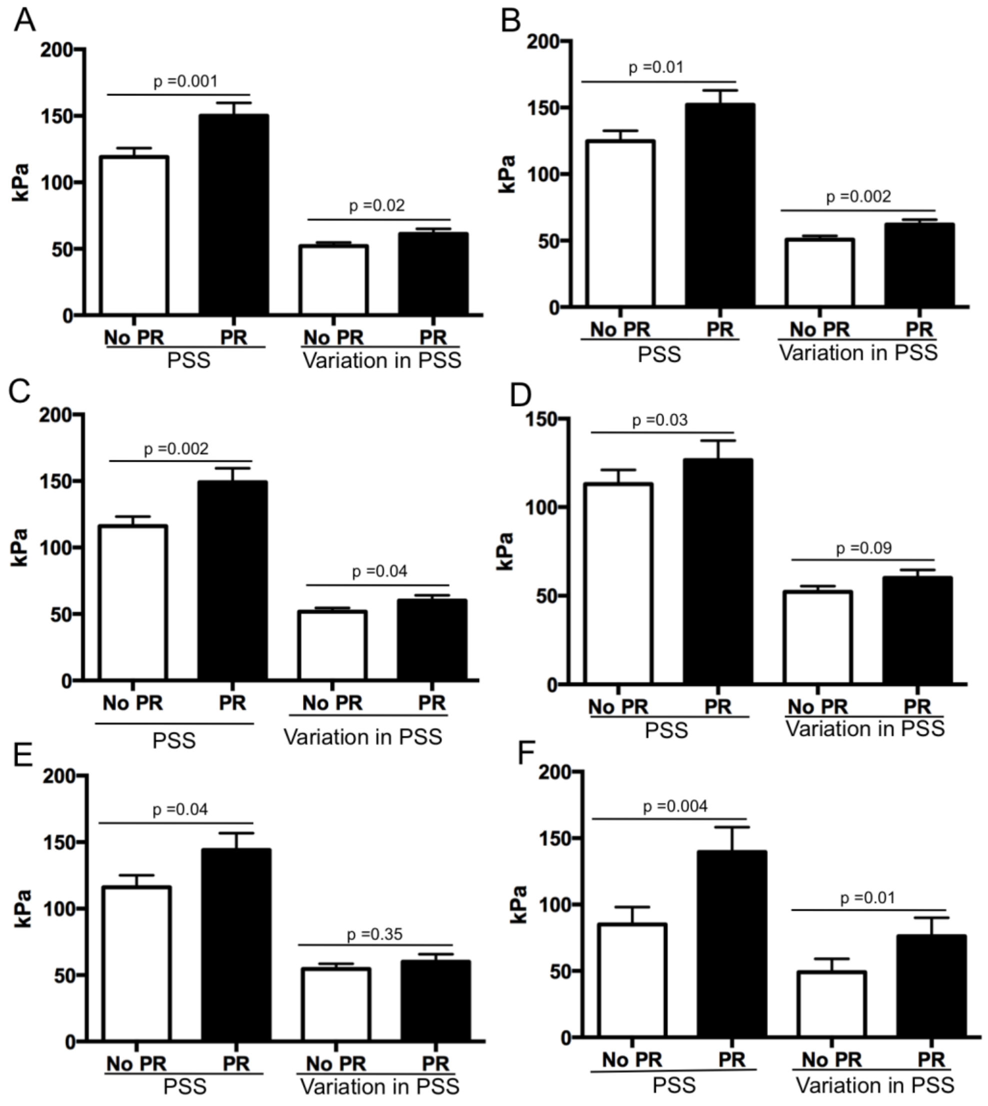

Figure 5. Variability of PSS both within and between plaques PSS from 6 consecutive VH-IVUS frames in 4 (A-D) different plaques.

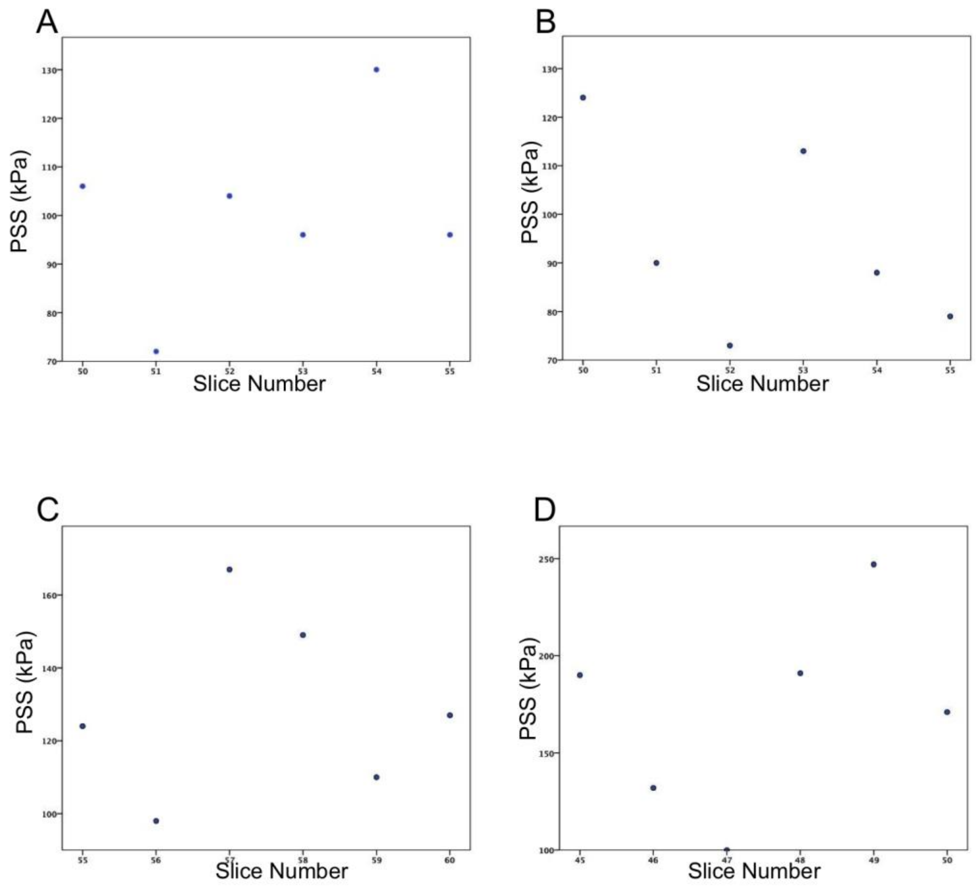

Figure 6. Distribution graphs for (A) PSS and (B) variation in PSS in the proximal and distal segments of ruptured plaque.

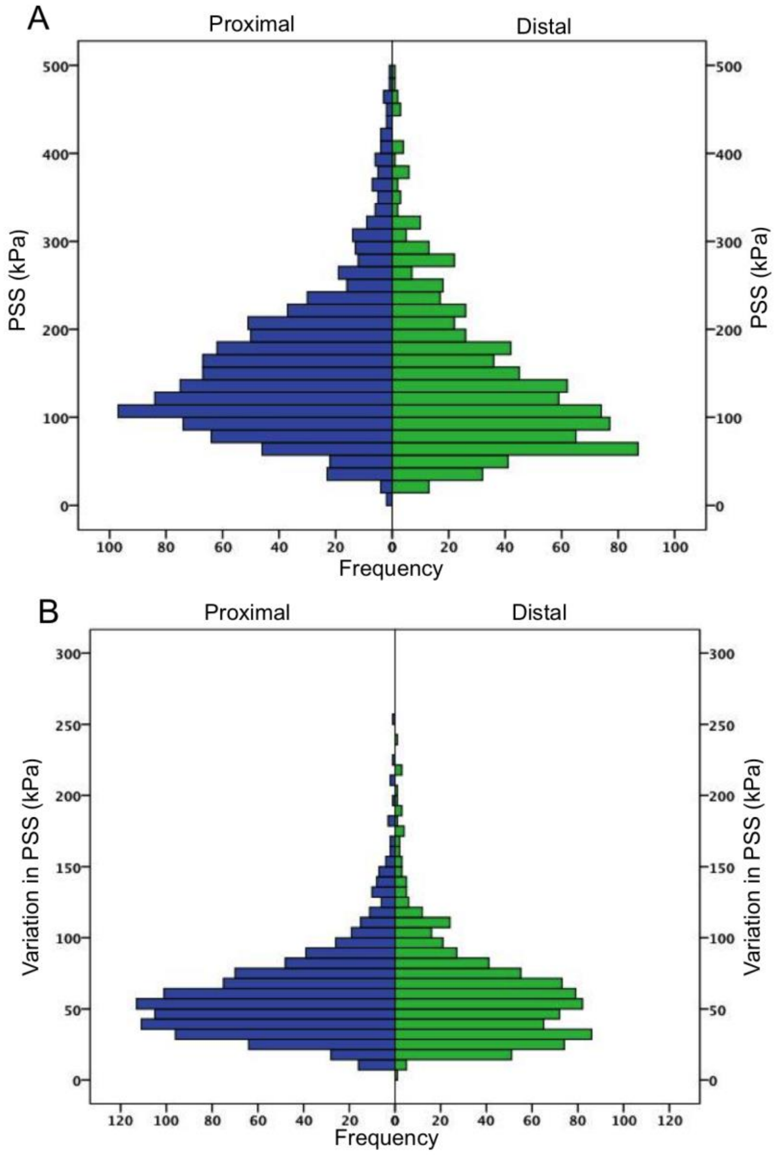

Tables

Table 1. Demographics of patients with ruptured and non-ruptured VH-TCFAs

Characteristic	Ruptured (n=23)	Non-ruptured (n=24)	p-value
Age, y	61.7 (10.7)	63.7 (9.8)	0.68
Male, n (%)	19 (82.6)	22 (91.7)	0.44
Diabetes mellitus, n (%)	2 (9)	3 (12.5)	0.37
Hypertension, n (%)	8 (35)	5 (20.8)	0.11
Hypercholesterolemia, n (%)	7 (30)	4 (16.7)	0.10
Smoker, n (%)	7 (30)	4 (16.7)	0.10
Family history of CAD, n (%)	13 (57)	12 (37.5)	0.33
Procedural blood pressure			
Systolic blood pressure, mm Hg	131.2 (25.3)	123.7 (19.9)	0.26
Diastolic blood pressure, mm Hg	67.4 (8.1)	66.5 (9.6)	0.84
Total cholesterol, mmol/L	3.63 (0.91)	3.85 (1.0)	0.50
LDL cholesterol, mmol/L	2.39 (0.89)	2.74 (1.12)	0.29
HDL cholesterol, mmol/L	1.24 (0.50)	1.1 (0.3)	0.28
Total cholesterol:HDL ratio	3.3 (1.72)	3.81 (1.79)	0.40
Creatinine, mg/DL	86.5 (17.8)	97.6 (24.1)	0.09
hsCRP, mg/L	11.8 (30.2)	6.0 (9.9)	0.38

Data are shown as mean (SD) or n (%). CAD=coronary artery disease; hsCRP=high-sensitivity C-reactive protein; HDL=high-density lipoprotein; LDL=low-density lipoprotein; MI=myocardial infarction; VH-TCFA=virtual-histology thin-cap fibroatheroma

Table 2. VH-IVUS parameters of ruptured and non-ruptured VH-TCFAs

Characteristic	Ruptured (n=29)	Non-Ruptured (n=24)	p-value
Plaque burden, (%)	60.1 (6.9)	62.9 (4.0)	0.12
Minimal luminal area ≤4mm^2, n (%)	29 (100)	24 (100)	NA
Fibrous/fibrofatty, (%)	59.8 (53-68)	65.1 (55-72)	0.16
Necrotic core, (%)	19.5 (17-28)	20.1 (16-23)	0.87
Dense calcium, (%)	16.1 (12-24)	14.5 (10-22)	0.32
Plaque characteristics at frame level			
Fibrous/fibrofatty, (%)	65.3 (48-80)	66.3(48-81)	0.25
Necrotic core, (%)	17.8 (11-28)	17.1 (10-29)	0.53
Dense calcium, (%)	12.7 (5-25)	11.8 (5-25)	0.21
Maximum arc of fibrous/fibrofatty tissue, (°)	134.6 (92-200)	136.3 (92-203)	0.23
Maximum arc of necrotic core, (°)	40.8 (27-62)	39.5 (24-60)	0.23
Maximum arc of dense calcium, (°)	43.8 (21-80)	37.6 (19-64)	0.01
Lumen eccentricity	0.51 (0.4-0.6)	0.50 (0.4-0.6)	0.61

Data are shown as mean (SD) or n (%). VH-TCFA=virtual-histology thin-cap fibroatheroma

Table 3. Correlation coefficients between variability in PSS and VH-IVUS parameters for VH-TCFAs

Characteristic	Correlation coefficient (r)	p-value
Luminal area, (mm^2)	0.23	0.001
Plaque burden, (%)	-0.12	0.001
Lumen eccentricity	0.06	0.001
Necrotic core ≥10, (%)	0.06	0.001
Maximum arc of necrotic core, (°)	0.01	0.55
Dense calcium ≥10, (%)	0.05	0.04
Maximum arc of dense calcium, (°)	0.007	0.69
Fibrous/fibrofatty tissue, (%)	-0.08	0.001
Maximum arc of fibrous/fibrofatty tissue, (°)	-0.16	0.001

PSS=plaque structural stress; VH-IVUS=virtual-histology intravascular ultrasound; VH-TCFA=virtual-histology thin-cap fibroatheroma

References

1. Calvert PA, Obaid DR, O'Sullivan M, et al. Association between IVUS findings and adverse outcomes in patients with coronary artery disease: the VIVA (VH-IVUS in Vulnerable Atherosclerosis) Study. J Am Coll Cardiol Img 2011;4:894-901.

2. von Birgelen C, Klinkhart W, Mintz GS, et al. Plaque distribution and vascular remodeling of ruptured and nonruptured coronary plaques in the same vessel: an intravascular ultrasound study in vivo. J Am Coll Cardiol 2001;37:1864-70.

3. Rodriguez-Granillo GA, Garcia-Garcia HM, Valgimigli M, et al. Global characterization of coronary plaque rupture phenotype using three-vessel intravascular ultrasound radiofrequency data analysis. Eur Heart J 2006;27:1921-7.

4. Ozaki Y, Okumura M, Ismail TF, et al. Coronary CT angiographic characteristics of culprit lesions in acute coronary syndromes not related to plaque rupture as defined by optical coherence tomography and angioscopy. Eur Heart J 2011;32:2814-23.

5. Fukumoto Y, Hiro T, Fujii T, et al. Localized elevation of shear stress is related to coronary plaque rupture: a 3-dimensional intravascular ultrasound study with in-vivo color mapping of shear stress distribution. J Am Coll Cardiol 2008;51:645-50.

6. Tang D, Teng Z, Canton G, et al. Local critical stress correlates better than global maximum stress with plaque morphological features linked to atherosclerotic plaque vulnerability: an in vivo multi-patient study. Biomed Eng Online 2009;8:15.

7. Huang Y, Teng Z, Sadat U, et al. Non-uniform shrinkage for obtaining computational start shape for in-vivo MRI-based plaque vulnerability assessment. J Biomech 2011;44:2316-9.

8. Teng Z, Zhang Y, Huang Y, et al. Material properties of components in human carotid atherosclerotic plaques: a uniaxial extension study. Acta Biomater 2014;10:5055-63.

9. Ebenstein DM, Coughlin D, Chapman J, Li C, Pruitt LA. Nanomechanical properties of calcification, fibrous tissue, and hematoma from atherosclerotic plaques. J Biomed Mater Res A 2009;91:1028-37.
